# Phubbing and phubber behavior: A new perspective in clinical psychological assessment

**DOI:** 10.3934/publichealth.2025037

**Published:** 2025-07-15

**Authors:** Carmela Mento, Maria Catena Silvestri, Clara Lombardo, Amelia Rizzo, Fabrizio Turiaco, Maria Rosaria Anna Muscatello, Fabio Presaghi

**Affiliations:** 1 Department of Biomedical and Dental Sciences and Morphofunctional Imaging, University of Messina, Messina, Italy; 2 University of Messina, Messina, Italy; 3 Department of “Scienze della Salute”, University of Catanzaro, Catanzaro, Italy; 4 Department of Psychology, University La Sapienza, Rome, Italy

**Keywords:** phubbing behavior, phubber behavior, psychological assessment, nomophobia, internet addiction, mental health

## Abstract

**Background:**

The term “Phubbing” has been defined as a behavior in which a person snubs another in a social setting by focusing on their phone instead of having a conversation. Phubbing is a common phenomenon and reduces the quality of social interactions in people, particularly those in adolescence.

**Objective:**

We aimed to validate in Italian, the Generic Scale of Phubbing (GSP), and Generic Scale of Being Phubbed (GSPB), in order to measure the experiences of phubbing, and being phubbed, through confirmatory factor analysis (CFA).

**Methods:**

We investigated whether the factor structure of GSP and GSBP may be replicated for the Italian sample, through confirmatory factor analysis (CFA). For the assessment, we used the Generic Scale of Being Phubbed, the Generic Scale of Phubbing, the Internet Addiction Test, and Brief COPE.

**Results:**

We found that four important factors of phubbing are nomophobia, interpersonal conflict, self-isolation, and acknowledgement of problems, and phubbing behavior is predictive of social disconnectedness.

**Conclusion:**

The GSP and GSBP instruments can be useful in the clinical setting to identify specific psychological dimensions associated with phubbing, such as nomophobia and social isolation.

## 1. Introduction

The term “phubbing” was introduced in Australia in 2007 [1] and made its first appearance in an advertising campaign promoted by the Macquarie Dictionary. In May 2012, a Melbourne-based advertising agency launched a campaign to combat this behavior, inviting linguists, writers, and poets to coin a word to describe it. From this effort, the term “phubbing” was created, a fusion of “phone” and “snubbing” [2]. The term later gained widespread global media coverage and became popular through the “Stop Phubbing” campaign created by McCann [3].

Few researchers have thoroughly explored the causes and mechanisms through which this behavior has become an accepted or normative feature of modern communication [4]. Understanding how people practice and experience phubbing is crucial to explaining this change. In social psychology, the concept of reciprocity helps to clarify how behavior is imitated or reciprocated, contributing to its normalization [5]. When phubbing is reciprocated between people, it tends to reinforce and consolidate as habitual behavior, progressively influencing the perception that it is normal or acceptable [6]. Over time, these dynamics can make phubbing a shared social norm, influenced by both observable and personal behavior. However, the consequences of smartphone use on the quality of social interactions have garnered increasing interest from researchers. In particular, the study by Dwyer and collaborators [7] highlighted that the presence of smartphones can compromise the quality of interpersonal interactions, as people tend to avoid face-to-face contact, thus losing essential communication skills.

Misra and colleagues [8] found that conversations where a smartphone was present showed lower levels of empathy compared to those where the device was absent, resulting in a perceived decrease in relationship quality and lower trust in the partner [9],[10]. The effects of phubbing can generate negative emotional responses, with individuals perceiving a lower quality of interactions, reduced relational satisfaction [11], and diminished trust in the partner [12]. Moreover, these dynamics can lead to feelings of jealousy [8],[13] and increase the risk of developing depressive moods [10]. This phenomenon has increased significantly in recent years, and it should be noted that phubbing is more common than has been thought in the past, and its possible effects can be more pervasive; the growing use of technology has entailed various problems, such as technology addiction [14],[15]. Interestingly, the study of Lo Coco and colleagues explored the relationship between Fear of Missing Out (FoMO) and problematic smartphone use (PSU) among adolescents and young adults. The authors found that FoMO and PSU are positively correlated [16]. This data indicates that excessive smartphone use could be related to the fear of missing out on something online, thus leading to constant social checking, for example. This is in line with a large body of research, which has reported that phubbing can be seen as a serious mental health problem for several reasons. It can cause symptoms like those substance addiction, including overuse, tolerance, withdrawal, and disturbances in daily life; moreover, preventing a person from using their smartphone elicits symptoms of tension, restlessness, and deprivation, and this pattern of response shows similarities with withdrawal symptoms seen in substance use disorders. Furthermore, phubbing is also associated with low self-esteem, behavioral and emotional difficulties, and poor communication among adolescents [17]. Ang et al. [18] explored the relationship between problematic use of smartphones, communication disturbances, and social connectedness in a sample of adolescents. Results confirmed that phubbing behavior was predictive of social disconnectedness. Despite the growing research interest in phubbing and phubbed behavior, very few measures assessing such behavior exist to date. Potential candidate questionnaires include the Generic Scale of Phubbing (GSP) and the Generic Scale of Being Phubbed (GSBP) [19]. The Generic Scale of Phubbing (GSP) is a tool designed to measure phubbing behavior in social interactions, assessing four factors: Nomophobia (NP), reflecting the fear of being disconnected from one's phone; interpersonal Conflict (IC), referring to perceived tensions between oneself and others due to phone use; self-Isolation (SI), describing the tendency to withdraw from social activities and seek solitude through phone engagement and Problem Acknowledgment (PA), indicating the recognition of a phubbing-related issue.The Generic Scale of Being Phubbed (GSBP) is an instrument that measures how often a person feels ignored in social interactions due to others' use of smartphones. The GSBP includes a series of items that explore different social situations in which phubbing may occur, aiming to quantify the impact of this behavior on the relational and psychological well-being of those who experience it. The GSBP was composed of three factors: Perceived Norms (PN), represented individuals' perceptions of how others use their phones; feeling Ignored (FI), indicated the feeling of being neglected because of others' phone use; and IC, reflected the perceived tension and disagreements arising from cell phone use in social interactions. The resulting scores help assess how frequently a person feels ‘phubbed’ and what emotional or social effects arise from it, such as feelings of exclusion, frustration, and dissatisfaction in interpersonal relationships. These tools are particularly useful for understanding the emotional and relational consequences of this phenomenon, contributing to exploring the dynamics of psychological distress and relational communication [20]. Phubbing is also assessed with other instruments, including The Phubbing Scale (PS) [2] and The Partner Phubbing Scale (Pphubbing) [10]. The PS includes 10 items on a five-point scale, from 1 (never) to 5 (always), to assess the degree to which individuals are distracted by their interlocutors, immersed in their phones, and tend to avoid social interactions. The Phubbing, unlike the other scales, assesses phubbing behavior in the context of romantic relationships. It consists of 9 items on a five-point scale, from 1 (never) to 5 (always), which determine the extent to which an individual's romantic partner uses or is distracted by their mobile phone during their time together. The GSP and GSBP scales are available in English, Spanish, Turkish, Chinese, Lebanese, and Peruvian [19],[21]–[25]. To date, there are no adaptations of the GSP and GSBP for use in the Italian context. We aimed to develop and validate an Italian version of the Generic Scale of Phubbing (GSP) and Generic Scale of Being Phubbed (GSBP) in a confirmatory factor analysis (CFA).

## 2. Materials and methods

### 2.1. Data collection and procedures

The four psychological instruments, the GSP, GSBP, the Internet Addiction Test (IAT), and the Brief COPE, were integrated into an online data collection platform (e.g., Google Forms®) and distributed through institutional mailing lists, social media posts on platforms such as Facebook® and LinkedIn®, additional professional mailing lists, and online advertisements.

To ensure data completeness, the online platform required participants to answer all questions before proceeding, preventing incomplete submissions. Informed consent, which briefly outlined the study's objectives and guaranteed anonymity, was presented at the beginning of the web page. All procedures for online data collection were conducted in full compliance with the General Data Protection Regulation (GDPR), ensuring the protection of participants' personal data, anonymity, and informed consent. Only fully completed questionnaires were considered valid for analysis. Participants took approximately 10–20 minutes to complete the survey, and smartphone use was permitted. The study was conducted with healthy subjects in accordance with the Helsinki Declaration. Ethical approval was not necessary.

### 2.2. Participants

A sample of 729 participants (Male *N* = 213; Female *N* = 516) with an age ranging from 12 to 76 years old (y.o.) with an average age of *M* = 32.96 (*SD* = 13.07) and a Median age of Me = 28 y.o (Inter-quartile Interval: 25° = 24 y.o.; 75° = 41 y.o.). One participant did not report their age. No differences emerged between age of Male and Female participants (Student *t* (727) = 0.67, *p* = 0.51; Male *M* = 33.17, *SD* = 13.17, *Min*-*Max* = 12–76; Female *M* = 32.46, *SD* = 12.75, *Min*-*Max* = 16–72). At the time of completing the survey, about 46.2% of the sample (*N* = 337) declared to be employed, about 40.7% to be students (*N* = 297), and the remaining participants declared to be unoccupied (11.6, *N* = 85) or retired (1.5%, *N* = 11). Moreover, about 68.6% of the sample (*N* = 501) reported to be not married at the moment of completing the survey, while about 26.7% of the sample (*N* = 195) was married and only 4.7% (*N* = 34) was divorced.

## 3. Measures

All participants included in the sample completed the following list of Questionnaires:

1. The GSP [19]. The scale contains 15 items and is measured at a seven-point Likert scale (1: Never, 7: Always). The scale consists of four subscales: NP, IC, SI, and PA.

2. The GSBP [19], the scale contains 22 items and is measured at a seven-point Likert scale (1: Never, 7: Always). The scale contains three subscales: PN, FI, and IC.

3. Internet Addiction Test (IAT) [26],[27]. The 20-items IAT were rated on a five-point scale (1 = rarely, 5 = always; McDonald' omega = 0.929, *M* = 39.05, *SD* = 12.56), assesses Internet addictive behavior based on the DSM-IV criteria (Diagnostic and Statistical Manual of Mental Disorder, 4th Edition) for pathological gambling and alcoholism such as “How often do you feel preoccupied with the Internet when off-line, or fantasize about being-online?” and “How often do you choose to spend more time on-line over going out with others?”. The IAT was used because, in addition to measuring Internet addiction in a broad sense, it also contains dimensions for the analysis of dysfunctional behavior related to the use of the device in social contexts.

4. Brief COPE [28] includes 14 scales, 8 of which measure presumably adaptive coping strategies and 6 of which focus on presumably maladaptive coping. Each of the 14 scales is captured by two items, and responses are made on 4-point scales (1–I have not been doing this at all; 4–I have been doing this a lot). Reliability estimates ranged from a minimum of 0.469 (Denial) to a maximum of 0.882 (Substance Use). Low reliabilities of some dimensions are in line with those reported in the original article [29].

### 3.1. Translation, back translation of the GSP, and GSBP

The GSP and GSBP were developed using the back-translation method. The items were initially translated from English to Italian by two independent translators, who produced two separate versions of the questionnaire. These versions were then integrated to create a unified version. Finally, two additional translators translated the Italian version back into English to ensure its equivalence with the original version.

## 4. Statistical analysis

We investigate whether the factor structure of the Italian version of the GSP and of the GSBP may be replicated on the Italian sample. Descriptive statistics (*M*, *SD*, Skewness, and Kurtosis), histograms, and Shapiro-Wilk tests for normality of distribution for each item are performed. Moreover, reliability and validity coefficients for each test are calculated.

## 5. Results

Descriptive statistics (*M*, *SD*, Skewness, and Kurtosis), histograms and Shapiro-Wilk tests for normality of distribution for each item are reported in Tables S1–S4 and in Figures S1–S4 of the supplementary materials.

### 5.1. Model comparison for the Italian version of the GSP

In Table 1, we compare models and fit indices of validated and published versions of GSP and GSBP. In the original version of the GSP and GSBP [16], the authors tested a one-factor second-order model with 4 first-order factors for both GSP and GSBP and compared fit indices to the corresponding unidimensional model. Also, a version with correlated errors was provided in the attempt to improve the models' fit. Ruiz et al. [19] and Kaya et al. [20] tested a similar model (but with a different number of d.f.), Li [18], and Bitar et al. [21] tested a four-factor first-order correlated factor structure.

**Table 1.** Models and corresponding fit indices of Validated versions of GSB and GSBP.

Study	*Language*	*Measure*	*Model*	*N*	*C2*	*df*	*p*	RMSEA	CFI	TLI
Chotpitayasunondh, and Douglas (2018)	ENG	GSP	Second Order Model	333	260.36	86	<0.001	0.08	0.95	0.93
Chotpitayasunondh, and Douglas (2018)	ENG	GSP	Second Order Model*	333	184.37	84	<0.001	0.06	0.97	0.95
Chotpitayasunondh, and Douglas (2018)	ENG	GSBP	Second Order Factor Model	341	783.77	206	<0.001	0.09	0.92	0.90
Chotpitayasunondh, and Douglas (2018)	ENG	GSBP	Second Order Factor Model*	341	433.46	198	<0.001	0.06	0.97	0.94
Ríos Ariza et al. (2021)	PER	GSP	Unspecified Model	454	222.00	-	-	0.057	0.91	-
Li (2023)	CHI	GSP	Four First Order Factors Model*	907	150.36	51	<0.001	0.05	0.99	0.98
Li (2023)	CHI	GSBP	Three First Order Factors Model*	907	1502.38	168	<0.001	0.09	0.95	0.93
Bitar et al. (2023)	LEB	GSP	Four First Order Factors Model	461	181.74	84	--	0.076	0.94	0.92
Ruiz et al. (2024)	SPA	GSP	Second Order Model*	346	171.246	84	<0.001	0.063	0.950	0.937
Ruiz et al. (2024)	SPA	GSP	Second Order Model**	346	152.598	83	<0.001	0.057	0.960	0.949
Kaya et al. (2024)	TUR	GSP	Second Order Model (sample a)	206	-	-	-	0.084	0.934	0.913
Kaya et al. (2024)	TUR	GSP	Four First Order Factors Model (sample a)	206	-	-	-	0.060	0.968	0.956
Kaya et al. (2024)	TUR	GSP	Second Order Model (sample b)	245	-	-	-	0.079	0.933	0.912
Kaya et al. (2024)	TUR	GSP	Four First Order Factors Model (sample b)	245	-	-	-	0.064	0.957	0.941
Kaya et al. (2024)	TUR	GSBP	Three First Order Factors Model (sample b)	206	-	-	-	0.051	0.975	0.968
Kaya et al. (2024)	TUR	GSBP	Second Order Factor Model (sample b)	245	-	-	-	0.064	0.975	0.968

Note: * two residual covariances added to the second order factor model to improve the fit; ** The authors modified the second order factor model to improve the fit

For the GSP model, we first compared the fit indices of different competing models, and in particular the two GSP competing models: The correlated first-order model and the one second-order factor model with first-order factors are compared to the one factor model and a three-factor model where we collapsed the NP and PA items into one factor. To choose the best fitting GSBP model, we also compared the fit indices of different models: The correlated first order factor model and the one second-order factor with the first order correlated factors model with the one factor model. Successively, we estimate Average Variance Extracted (AVE) and Composite Reliability (CR, or the McDonald' omega) for the final model to address discriminant validity properties. Finally, we computed deattenuated correlations among GSP and GSBP factors with IAT and COPE dimensions to address convergent and divergent validity issues.

Considering that items included in both GSP and GSBP consider behaviors that may be problematic and that it not assumed to be normally distributed in the generic population, we consider Robust Maximum Likelihood estimator with Satorra-Bentler fit statistics for fitting and evaluating Confirmatory Factor Models (CFA) that has been shown to perform optimally also under strong violation of the normality assumption [26]. Descriptive statistics (*M*, *SD*, Skewness, and Kurtosis as well as Shapiro-Wilk test for normality of distribution are available in the supplementary materials: Tables S1–S4 and Figures S1–S4). To assess goodness of fit of the models, we not only consider the fit statistics of each model, i.e., non-significant χ^2^, RMSEA (below 0.08), NLI, and CFI (both above 0.95). Due to the unbalance of gender groups, we cannot investigate the invariance of factor structure across gender. Composite McDonald's omega reliability indices [30] are also assessed for each estimated factor. Deattenuated correlations among GSP and GSBP factors are also considered for assessing construct validity of the two questionnaires. To address Convergent and Divergent validities of GSP and GSBP factors with IAT and COPE dimensions, we estimated deattenuated correlation coefficients. For the analysis, we used the R software [31], and for fitting all Confirmatory models, we used the lavaan package [30], [version 0.6–19] [32] by setting the maximum number of iterations and convergence criterion to the default (respectively: 20,000 and 1e–08).

**Figure 1.**
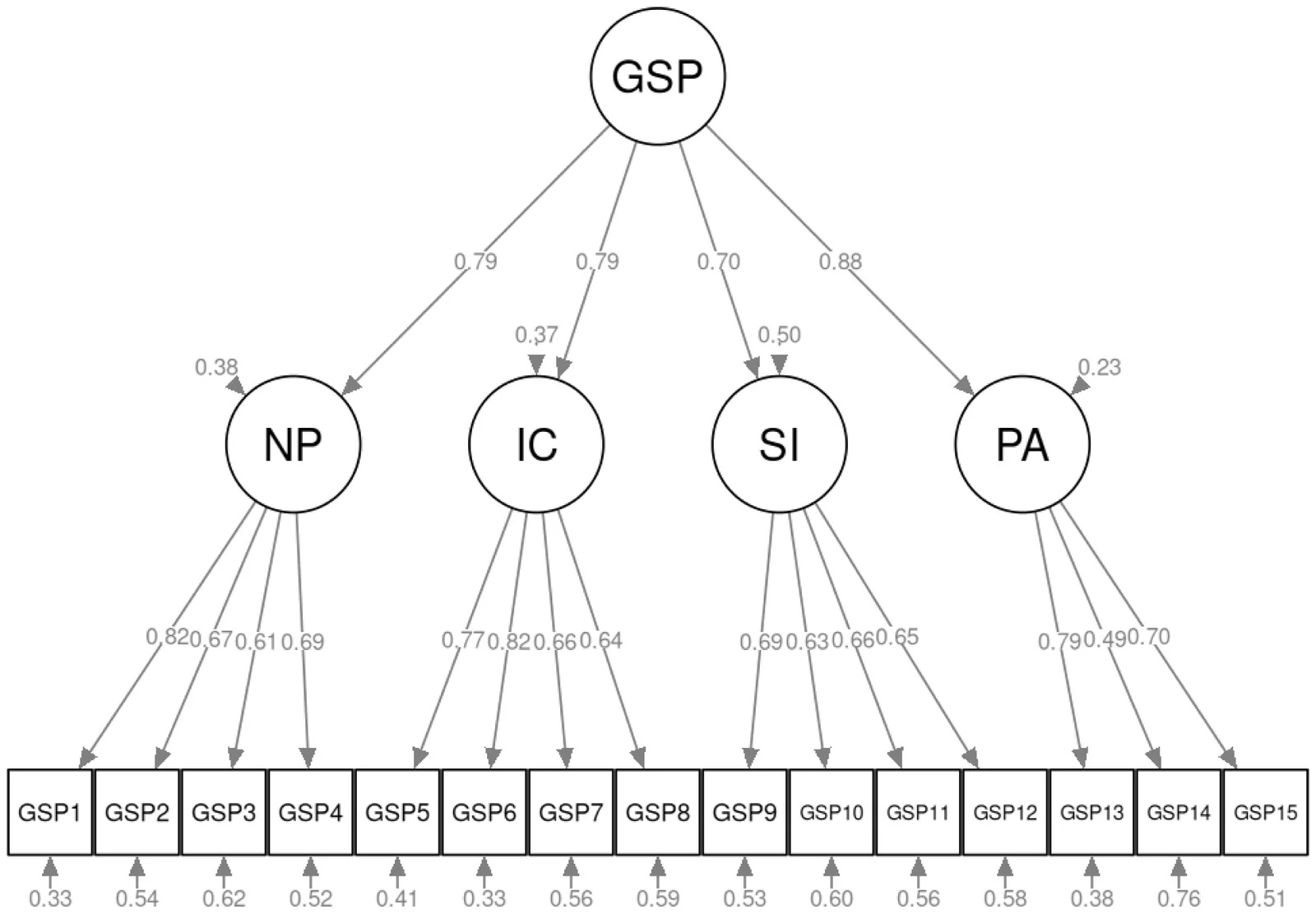
Path diagram of the GSP second-order with four first-order factors model.

As shown in Table 2, both the four-factor (*M1*) and the second order (*M2*) models of GSP reported good fit statistics compared to the one factor and to the three-factor models. The fit difference between the two major models (*M1*–*M2*) was not significant (Satorra-Bentler *Δχ^2^* (2) = 5.55, *p* = 0.06) meaning that the two models are equivalent, and we should prefer the model with less parameters, i.e., the second order model. Table 3 shows the robust parameter estimates (standardized and unstandardized factor loadings, *s.e*., and the 95% *C.I*.) for the second-order GSP model (Figure 1). Table S5 in the supplementary materials shows the residuals of 15 manifest GSP variables.

**Table 2.** Comparison of fit indices among GSP models.

Model	*χ^2^*	*df*	*p*	*RMSEA*	*RMSEA 90% C.I*.		CFI	TLI	Model compared	*Δχ^2^*	*Δdf*	*Δp*
					*LL*	UL						
[M1] Four-correlated first-order factors model	222.76	84	0	0.055	0.047	0.064	0.944	0.93	-	-	-	-
[M2] One second-order factor with four first-order factors model	228.24	86	0	0.055	0.047	0.064	0.943	0.93	*M1*–*M2*	5.55	2	0.062
[M3] Three-first factors model	341.71	87	0	0.074	0.065	0.082	0.904	0.884	*M2*–*M3*	111.45	1	0
[M4] One-factor model	735.73	90	0	0.116	0.108	0.124	0.764	0.725	*M2*–*M4*	404.44	4	0

**Table 3.** Robust estimates of unstandardized (*b*) and standardized (*λ*) factor loadings, *s.e*., and 95% *C.I*. for the Italian version of the 15 GSP items and first-order factors.

*Item* *Factor*	*Latent* *Factor*	*Unstandardized* *lambda*	*s.e.* *unstandardized* *lambda*	95% C.I.		Completely standardized lambda
				LL	UL	
GSP1	NP	0.84	0.06	0.72	0.96	0.82
GSP2	NP	0.75	0.06	0.63	0.88	0.67
GSP3	NP	0.68	0.06	0.57	0.79	0.61
GSP4	NP	0.75	0.06	0.64	0.86	0.69
GSP5	IC	0.61	0.05	0.52	0.71	0.77
GSP6	IC	0.78	0.05	0.67	0.88	0.82
GSP7	IC	0.47	0.04	0.39	0.55	0.66
GSP8	IC	0.45	0.04	0.36	0.53	0.64
GSP9	SI	0.50	0.05	0.40	0.60	0.69
GSP10	SI	0.36	0.04	0.28	0.45	0.63
GSP11	SI	0.48	0.05	0.37	0.59	0.66
GSP12	SI	0.60	0.06	0.50	0.71	0.65
GSP13	PA	0.68	0.09	0.51	0.86	0.79
GSP14	PA	0.37	0.05	0.26	0.48	0.49
GSP15	PA	0.61	0.08	0.45	0.77	0.70
NP	GSP	1.29	0.13	1.04	1.54	0.79
IC	GSP	1.30	0.12	1.06	1.54	0.79
SI	GSP	0.99	0.10	0.79	1.19	0.70
PA	GSP	1.85	0.29	1.29	2.41	0.88

Note: NP = Nomophobia; IC = Interpersonal Conflict; SI = Self-Isolation; PA = Problem Acknowledgement; LL = Lower limit of the 95% *C.I*.; UL = Upper limit of the 95% *C.I*.; *N* = 730.

### 5.2. AVE, correlations, and CR of the Italian version of the GSP factors

Table 4 shows AVE, correlations, and CR for discriminant validity. All CR indices are higher than 0.70. Unfortunately, three of the GSP four first-order factors showed an AVE index slightly below the threshold of 0.50, which is the threshold for concluding that GSP factors have good convergent validity. Turning to the discriminant validity of the Italian version of GSP, as reported by Fornell and Larker [33], a latent factor to show a good discriminant validity should have the square root of the AVE index higher than the inter-construct correlations. As shown in Table 4, the square root of the lowest AVE index (0.660), reported by Self-Isolation, is greater than all inter-construct correlations, meaning that the four first-order GSP factors showed adequate discriminant validity. In conclusion, even if the slightly below cut-off AVE indices may be indicative of modest convergent validity issues, this is partly compensated by the adequate discriminant validity indices.

**Table 4.** Average Variance Extracted (AVE), correlations, and Composite Reliability (CR).

*Row*	*AVE*	*CR*	*NP*	*IC*	*SI*	PA	GSP
NP	0.488	0.794	1				
IC	0.552	0.825	0.506	1			
SI	0.435	0.752	0.418	0.494	1		
PA	0.467	0.719	0.528	0.552	0.473	1	
GSP	-	0.781	0.813	0.792	0.694	0.837	1

Note: NP = Nomophobia; IC = Interpersonal Conflict; SI = Self-isolation; PA = Problem Acknowledgement; Inter-construct Correlation values equal or greater than rho = 0.418 have a *p* < 0.01; *p*-values are adjusted for multiple comparisons using the Holm method.

### 5.3. Model comparison for the Italian version of the GSP

**Figure 2.**
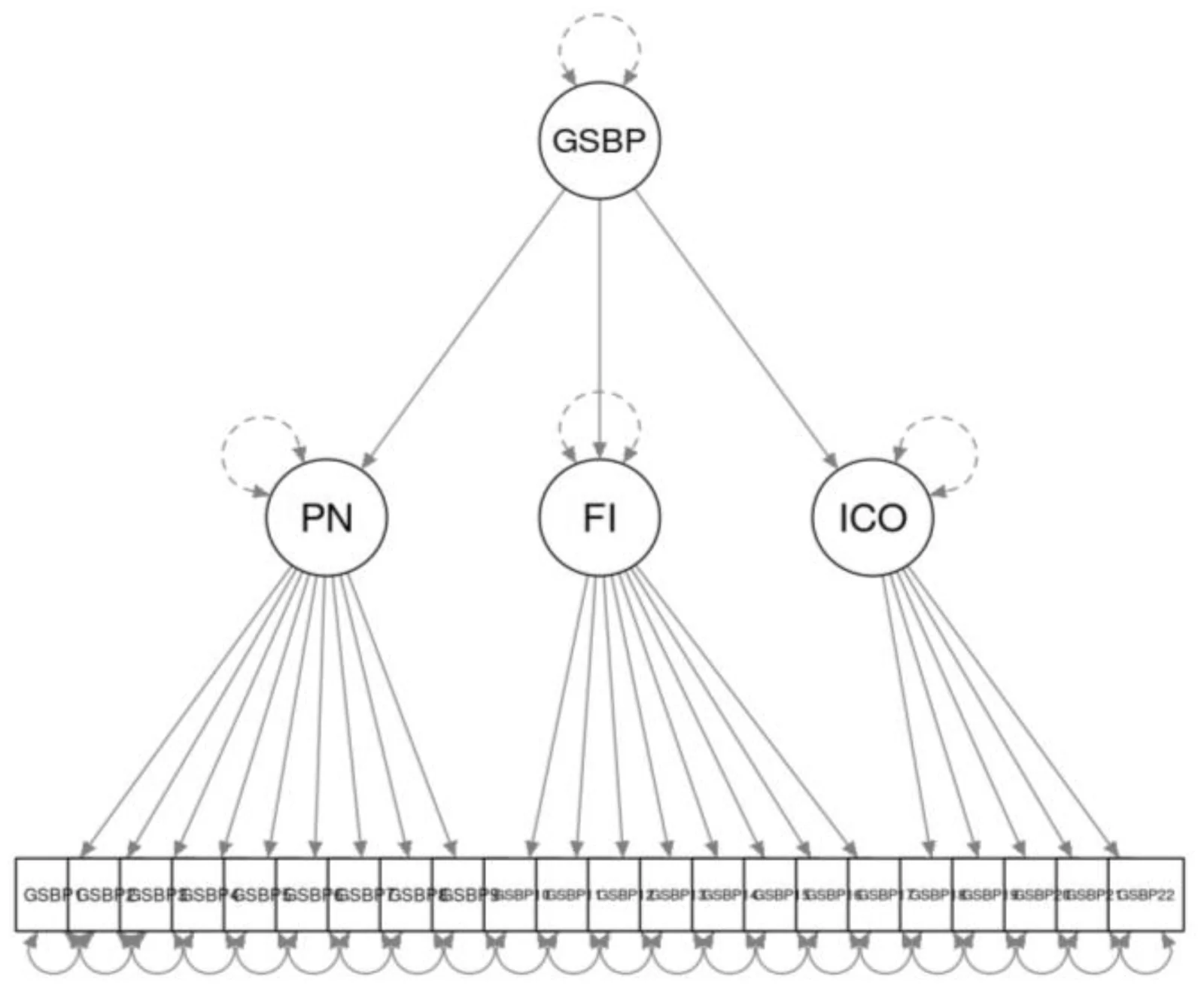
The GSBP path model.

In Table 5, we report the fit indices for the first-order factors (M1) and the second-order factor models (M2) of the Italian version of the GSBP. Fit indices of the two models were not satisfactory, reporting values of RMSEA above the cutoff of 0.08 and values of CFI and TLI below the cutoff of 0.90. Following the suggestions of Chotpitayasunondh and Douglas [19] who released some residual covariances between manifest variables, we improved the fit indices of our models (respectively, M1a and M2a; we freed the residual covariance of item GSBP1 and item GSBP2, and the residual covariance of item GSBP2 and item GSBP3). Having the same number of estimated parameters, the two models showed exactly the same fit, so in this case, we could not choose the model based on differential fit. However, for the sake of comparison with the original English version and the Turkish version of GSBP, we selected the second-order factor model (M2a). Finally, the M2a fit was strikingly better than the fit of the one-factor model. Table 6 shows the standardized and unstandardized factor loadings, the *s.e*., and the 95% *C.I*. for each item and first-order factors of the Italian version of the GSBP (Figure 2). Table S6 in the supplementary materials reports the residuals between the Italian version of the 22 GSBP items.

**Table 5.** Comparison of fit indices among models of the Italian version of the GSBP.

*Model*	*χ^2^*	*df*	*p*	*RMSEA*	*RMSEAS 90% C.I.*		CFI	TLI	Comparison	*Δχ^2^*	*Δdf*	*Δp*
					LL	UL						
[M0] One first-order factor model	3242.61	209	<0.01	0.157	0.152	0.162	0.658	0.622	-	-	-	-
[M1] Three correlated first-order factors model	1116.40	206	<0.01	0.088	0.083	0.093	0.89	0.877	M1–M0	NA	3	-
[M1a] Three correlated first-order factors model with covariances between Residuals	93.65	204	<0.01	0.079	0.074	0.084	0.911	0.900	M1–M1a	199.67	2	<0.01
[M2] One Second-order factor and 3 first order factors model	1116.40	206	<0.01	0.088	0.083	0.093	0.89	0.877	M2–M1a	199.68	2	<0.01
[M2a] One Second-order factor and 3 first order factors model with covariances between Residuals	93.65	204	<0.01	0.079	0.074	0.084	0.911	0.900	M2a–M1a	0	0	-

**Table 6.** Robust estimates of unstandardized (b) and standardized (λ) factor loadings, *s.e*., and 95% *C.I*. for each item and first-order factors of the Italian version of the GSBP.

*Item* *Factor*	*Latent* *Factor*	*Unstandardized* *lambda*	*s.e.* *unstandardized* *lambda*	*95% C.I.*		Completely standardized lambda
				*LL*	*UL*	
GSBP1	PN	0.45	0.06	0.35	0.56	0.36
GSBP2	PN	0.44	0.05	0.34	0.55	0.35
GSBP3	PN	0.53	0.05	0.44	0.62	0.51
GSBP4	PN	0.76	0.04	0.68	0.84	0.66
GSBP5	PN	0.84	0.05	0.75	0.93	0.74
GSBP6	PN	0.87	0.04	0.79	0.95	0.83
GSBP7	PN	0.90	0.04	0.81	0.98	0.80
GSBP8	PN	0.82	0.04	0.73	0.90	0.76
GSBP9	PN	0.88	0.04	0.79	0.96	0.75
GSBP10	FI	0.59	0.04	0.51	0.67	0.77
GSBP11	FI	0.66	0.04	0.58	0.75	0.85
GSBP12	FI	0.73	0.05	0.64	0.83	0.85
GSBP13	FI	0.70	0.05	0.60	0.80	0.84
GSBP14	FI	0.66	0.05	0.57	0.75	0.86
GSBP15	FI	0.68	0.05	0.59	0.77	0.81
GSBP16	FI	0.67	0.05	0.58	0.76	0.82
GSBP17	FI	0.59	0.04	0.51	0.67	0.75
GSBP18	ICON	0.67	0.05	0.56	0.77	0.75
GSBP19	ICON	0.69	0.05	0.58	0.79	0.82
GSBP20	ICON	0.78	0.06	0.67	0.89	0.85
GSBP21	ICON	0.71	0.05	0.61	0.82	0.85
GSBP22	ICON	0.70	0.05	0.60	0.81	0.77
PN	GSBP	0.89	0.07	0.75	1.03	0.66
FI	GSBP	1.51	0.14	1.23	1.79	0.83
ICON	GSBP	1.52	0.16	1.22	1.83	0.84

Note: PN = Perceived Norms; FI = Feeling Ignored; ICON = Interpersonal Conflict; LL = Lower Limit of the 95% *C.I*.; UL = Upper Limit of the 95% *C.I*.; *N* = 730.

### 5.4. AVE, latent correlations, and CR of the Italian version of the GSBP factors

Table 7 shows the AVE, the latent correlations, and the CR for the Italian version of the GSBP latent factors. Also in this case, we found that only two of three first-order factors showed good convergent validity, reporting AVE values greater than 0.50. However, all three first-order factors reported good discriminant validity, as the square root of the lowest AVE index (0.648, Perceived Norms) was greater than all inter-construct correlations.

**Table 7.** AVE, correlations, and CR for the Italian version of the GSBP.

*Row*	*AVE*	*CR*	*PN*	*FI*	*IC*	GSBP
PN	0.42	0.810	1.000			
FI	0.675	0.944	0.476	1		
IC	0.653	0.906	0.462	0.652	1	
GSBP	-	0.774	0.749	0.862	0.871	1

Note: PN = Perceived Norms; FI = Feeling Ignore; IC = Interpersonal Conflict; Inter-construct correlation values equal or greater than rho = 0.462 have a *p* < 0.01; *p*-values are adjusted for multiple comparisons using the Holm method.

### 5.5. Inter-construct deattenuated correlations among the Italian version of the GSP and of the GSBP factors

Table 8 shows the deattenuated inter-construct correlations among the Italian version of the GSP and GSBP factors. As shown in Table 3, overall, the four first-order factors of the Italian version of the GSP had modest if null latent correlations with the three GSBP factors. In particular NP, SI, and PA correlated positively and statistically significantly with PN and FI, and with the exclusion of PA, the IC factor was uncorrelated with NP, IC, and SI factors of the GSP. The two Total indices correlated positively and significantly, but the low value of the correlation was indicative of the fact that the two constructs measured different aspects of the Phubbing or that the Being Phubbed did not correlate with IC. In conclusion, the two-factor structures seemed to be very poorly correlated, as shown in Table 3.

**Table 8.** Deattenuated inter-construct correlations among the Italian version of the GSP and GSBP factors.

	***PN***	***FI***	***IC***	***GSBP***
NP	0.356***	0.211***	0.068	0.255***
IC	0.093	0.202***	0.036	0.141***
SI	0.208***	0.243***	0.055	0.210***
PA	0.392***	0.270***	0.139***	0.328***
GSP	0.349***	0.289***	0.098	0.302***

Note: PN = Perceived Norms; FI = Feeling Ignore; IC = Interpersonal Conflict; NP = Nomophobia; IC = Interpersonal Conflict; SI = Self-isolation; PA = Problem Acknowledgement; GSB = Total score on the GSP; GSBP = Total score on the GSBP; Inter-construct Correlation values equal or greater than rho = 0.139 have a *p* ≤ 0.0002 (***); *p*-values are adjusted for multiple comparisons using the Bonferroni method.

### 5.6. Deattenuated Correlations among the Italian version of the GSP and GSBP factors and IAT and COPE factors

Table 9 shows deattenuated correlations among GSP and GSBP factors and related constructs of IA and COPE. IAT scores correlated positively with GSP factors and, on average, these correlations were higher than those showed by GSBP factors, confirming the results that GSP factors, but not GSBP, were strongly affected by IAT. The relationships among COPE factors and GSP and GSBP were more complicated to disentangle, as no clear pattern emerged from Table 9. Behavioral Disengagement reported the highest, even if modest in entity, correlations with GSP factors, meaning that the more the tendency to phub, the more people tended to use “behavioral disengagement” to cope with phub. While Denial was more related to GSBP factors, meaning that the more people get phubbed, the more they tended to recur to denial to cope with stressful situations. Moreover, Using Emotional Support had a positive correlation with PA (*r* = 0.358, *p* < 0.01), so the tendency to acknowledge the problem of phubbing favored the attempt to expect more emotional support from others. Finally, Venting was correlated with NP (*r* = 0.332, *p* < 0.01), meaning that the more anxiety to separate from a mobile phone, the greater the need to express such anxiety by venting.

**Table 9.** Deattenuated correlations between GSP factors and IAT and COPE factors, with Bonferroni corrected C.I. for multiple comparisons (1–0.05/135 = 0.9996296).

	*NP*	*C.I*		*IC*	*C.I*		*SI*	*C.I*		*PA*	*C.I*		*GSP*	*C.I*		*PN*	*C.I*		*FI*	*C.I*		*IC*	*C.I*	
		*LL*	*UL*		*LL*	*UL*		*LL*	*UL*		*LL*	*UL*		*LL*	*UL*		*LL*	*UL*		*LL*	*UL*		*LL*	*UL*
IAT score	0.60	0.66	0.54	0.57	0.63	0.49	0.66	0.71	0.61	0.86	0.87	0.84	0.85	0.87	0.83	0.34	0.44	0.24	0.31	0.41	0.20	0.16	0.28	0.04
Positive Reframing	−0.06	0.06	−0.18	−0.07	0.06	−0.19	−0.02	0.10	−0.15	0.04	0.17	−0.08	−0.03	0.09	−0.16	0.10	0.23	−0.02	−0.04	0.09	−0.16	−0.00	0.12	−0.13
Self-Distraction	0.18	0.29	0.06	0.06	0.18	−0.07	0.04	0.17	−0.08	0.25	0.36	0.13	0.18	0.30	0.06	0.31	0.41	0.20	0.07	0.19	−0.06	0.16	0.28	0.04
Venting	0.33	0.43	0.22	0.12	0.24	−0.00	0.09	0.21	−0.04	0.29	0.39	0.17	0.28	0.39	0.17	0.18	0.29	0.06	0.10	0.22	−0.03	0.14	0.26	0.01
Using Instrumental Support	0.22	0.33	0.10	0.11	0.23	−0.02	0.06	0.19	−0.06	0.26	0.36	0.14	0.22	0.33	0.10	0.11	0.23	−0.01	0.01	0.14	−0.11	0.06	0.18	−0.06
Active Coping	−0.01	0.12	−0.13	−0.14	−0.01	−0.26	−0.11	0.02	−0.23	−0.03	0.10	−0.15	−0.08	0.05	−0.20	0.14	0.26	0.02	−0.14	−0.02	−0.26	−0.11	0.02	−0.23
Denial	0.21	0.33	0.10	0.23	0.34	0.12	0.11	0.23	−0.01	0.20	0.32	0.08	0.25	0.36	0.13	0.33	0.43	0.22	0.31	0.41	0.20	0.33	0.43	0.22
Religion	−0.07	0.05	−0.20	−0.02	0.10	−0.15	−0.06	0.06	−0.18	−0.07	0.06	−0.19	−0.07	0.05	−0.19	0.03	0.16	−0.09	0.05	0.17	−0.08	0.03	0.15	−0.10
Humor	0.06	0.18	−0.07	0.10	0.22	−0.02	0.14	0.26	0.02	0.08	0.20	−0.04	0.11	0.23	−0.01	0.11	0.23	−0.01	0.15	0.27	0.02	0.14	0.26	0.02
Behavioral Disengagement	0.19	0.30	0.07	0.27	0.38	0.15	0.30	0.41	0.19	0.35	0.45	0.24	0.34	0.44	0.23	0.12	0.24	−0.01	0.17	0.29	0.05	0.14	0.26	0.01
Using Emotional Support	0.24	0.35	0.13	0.13	0.25	0.01	0.10	0.22	−0.02	0.36	0.46	0.25	0.28	0.39	0.17	0.13	0.25	0.01	0.09	0.21	−0.04	0.11	0.23	−0.02
Substance Use	0.13	0.25	0.01	0.04	0.16	−0.08	0.15	0.27	0.03	0.14	0.26	0.02	0.14	0.26	0.02	0.08	0.20	−0.04	0.10	0.22	−0.02	0.08	0.20	−0.04
Acceptance	0.01	0.14	−0.11	−0.10	0.03	−0.22	−0.08	0.05	−0.20	−0.07	0.06	−0.19	−0.06	0.06	−0.19	0.16	0.28	0.04	0.01	0.14	−0.11	0.03	0.15	−0.10
Planning	−0.04	0.09	−0.16	−0.16	−0.04	−0.28	−0.10	0.03	−0.22	−0.08	0.04	−0.20	−0.11	0.01	−0.23	0.10	0.22	−0.03	−0.09	0.04	−0.21	−0.07	0.06	−0.19
Self-Blame	0.19	0.31	0.07	0.07	0.19	−0.06	0.10	0.22	−0.02	0.28	0.39	0.17	0.22	0.33	0.10	0.24	0.35	0.12	0.23	0.35	0.12	0.17	0.29	0.05

Note: NP = Nomophobia; IC = Interpersonal Conflict; SI = Self-Isolation; PA = Problem Acknowledgement; GSP = Total score on the GSP; PN = Perceived Norms; FI = Feeling Ignored; IC = Interpersonal Conflict; GSBP = Total score on the GSBP; IAT score = Internet Addiction score.

## 6. Discussion

The results of our study confirm the validity of the GSP as a multidimensional instrument to measure phubbing. The four-factor model demonstrated a better fit than the one-factor model, supporting the idea that phubbing is a complex behavior influenced by several psychological variables, including nomophobia and self-isolation. Confirmatory factor analysis showed that a four-factor model (Nomophobia, Interpersonal Conflict, Self-isolation, and Problem Recognition) had a better fit to the data than a one-factor model, suggesting that phubbing is influenced by a series of interconnected psychological variables. Indeed, nomophobia represents one of the predominant factors that fuel phubbing behavior, confirming previous research linking smartphone addiction to this phenomenon [34]. Similarly, GSBP showed that being ignored in favor of a smartphone is also a multidimensional phenomenon. The three major factors that emerged (Perceived Norms, Feeling Ignored, and Interpersonal Conflict) reflect how individuals perceive and respond to phubbing behavior from others. Feeling ignored is particularly significant, as literature suggests that social exclusion resulting from phubbing can generate emotional distress and damage mental health, leading to feelings of isolation and anxiety; also, the impulsivity and depressive brooding may influence phubbing behavior and problematic internet use, particularly in pandemic contexts [4],[35]. Furthermore, social norms can strongly influence individual behavior: If a person perceives that phubbing behavior is accepted or tolerated in their social group, they will be more likely to engage in such behavior, perpetuating the cycle of social exclusion [36]. The gender differences that emerged in the scores related to Perceived Norms and Interpersonal Conflict are in line with the data present in the literature. Women tend to be more sensitive to signals of social exclusion than men and tend to give greater value to interpersonal relationships, and consequently could react more negatively to the interruption of the emotional connection that phubbing entails [22],[37]. Additionally, women experience greater emotional distress in response to phubbing and tend to perceive social norms related to smartphone use differently than men [37]. Another moderate correlation that emerged in this study is that between phubbing and Internet addiction. Participants with higher scores on the IAT tended to show higher phubbing behaviors, confirming that problematic Internet use and smartphone addiction are significant predictors of phubbing [2]. The concept of phubbing has only recently emerged, and to date, the scientific literature on the subject remains limited. The results of this study suggest that the GSP and GSBP tools may be useful in clinical settings to identify specific psychological dimensions associated with phubbing, such as nomophobia and social isolation. This identification can guide targeted interventions to reduce dysfunctional smartphone use and improve interpersonal relationships. Moreover, it is useful to consider the role of emotional variables for a better understanding of phubbing-related behavior. Such behavior could imply a lack of sensitivity to others, and emotional intelligence (EI) could be considered a predictor of prosocial behavior. This ability could mitigate the tendency to ignore others in interactions [38],[39]. Furthermore, the application of these tools in educational settings can facilitate prevention programs among adolescents, promoting greater awareness of the use of technology and the social dynamics associated with phubbing.

## 7. Limitations and future directions

Although collecting data via online surveys is expected to eliminate the pressure on participants, the inability to inform participants about the purpose and significance of the study face-to-face can be considered a limitation. Another limitation consists in the impossibility to assess the test-retest strategy for the anonymous condition of the online survey of the participants. An additional limitation in this study concerns the cross-sectional design, based on data collected at a single point in time. This methodological approach does not enable changes over time to be observed or causal relationships to be established between the variables investigated. Future studies, with longitudinal or experimental designs, will be necessary to further investigate the direction and nature of the observed relationships. A further limitation concerns aspects of emotional intelligence or perceived social support, which are closely related to subjective well-being. This could open new research areas for the protective effects of these variables on the negative impact of obesity. These constructs could play a significant role in modulating the impact of phubbing, influencing the relational modes of individuals and their ability to deal with complex interpersonal situations. Including such variables in future studies could contribute to a broader understanding of the psychological mechanisms underlying phubbing. The clinical implications of this study concern the possibility of operating in the field of prevention of similar behaviors, in order to improve the quality of social relationships, of people, and prevent such forms of addiction.

## Use of AI tools declaration

The author declare he has not used Artificial Intelligence (AI) tools in the creation of this article.


