# Supplementary material for: Phubbing and phubber behavior: A new perspective in clinical psychological assessment
Source: AIMS Public Health. 2025 Jul 15;12(3):716–34. doi: 10.3934/publichealth.2025037 (PMC12538237; doi:10.3934/publichealth.2025037)
Supplement: Supplementary file 1 [file publichealth-12-03-037-s001.pdf]

AIMS Public Health

AIMS Public Health, 12(3): 716–734.
DOI: 10.3934/publichealth.2025037
Received: 01 April 2025
Revised: 19 May 2025
Accepted: 24 June 2025
Published: 15 July 2025

https://www.aimspress.com/journal/aimsph

Research article

Phubbing and phubber behavior: A new perspective in clinical psychological assessment

Carmela Mento[1], Maria Catena Silvestri[2], Clara Lombardo[3,*], Amelia Rizzo[2], Fabrizio Turiaco[1], Maria Rosaria Anna Muscatello[1] and Fabio Presaghi[4]

[1] Department of Biomedical and Dental Sciences and Morphofunctional Imaging, University of Messina, Messina, Italy
[2] University of Messina, Messina, Italy
[3] Department of "Scienze della Salute", University of Catanzaro, Catanzaro, Italy
[4] Department of Psychology, University La Sapienza, Rome, Italy

* **Correspondence:** Email: clara.lombardo@unicz.it; Tel: +393478184471.

Supplementary

Table S1. Descriptive statistics (*M*, *SD*, Skewness, and Curtosis) as well as Shapiro-Wilk normality test for GSP item.

Row	*M*	*SD*	*Skewness*	*Kurtosis*	*SW.test*	*p*.value
GSP1	3.33	1.67	0.36	–0.75	0.93	<0.001
GSP2	3.48	1.82	0.33	–0.94	0.92	<0.001
GSP3	4.50	1.80	–0.41	–0.89	0.92	<0.001
GSP4	3.17	1.78	0.48	–0.80	0.91	<0.001
GSP5	2.18	1.31	1.12	0.68	0.81	<0.001
GSP6	2.48	1.56	1.07	0.58	0.84	<0.001
GSP7	1.65	1.16	2.18	4.81	0.62	<0.001
GSP8	1.79	1.15	2.23	5.87	0.68	<0.001
GSP9	1.65	1.03	2.33	6.82	0.65	<0.001
GSP10	1.38	0.81	3.16	13.06	0.52	<0.001
GSP11	1.51	1.01	2.89	9.80	0.56	<0.001
GSP12	2.05	1.31	1.58	2.34	0.76	<0.001
GSP13	3.44	1.82	0.43	–1.04	0.89	<0.001
GSP14	2.29	1.59	1.61	1.93	0.75	<0.001
GSP15	3.01	1.84	0.76	–0.63	0.86	<0.001

Note: $N = 730$.

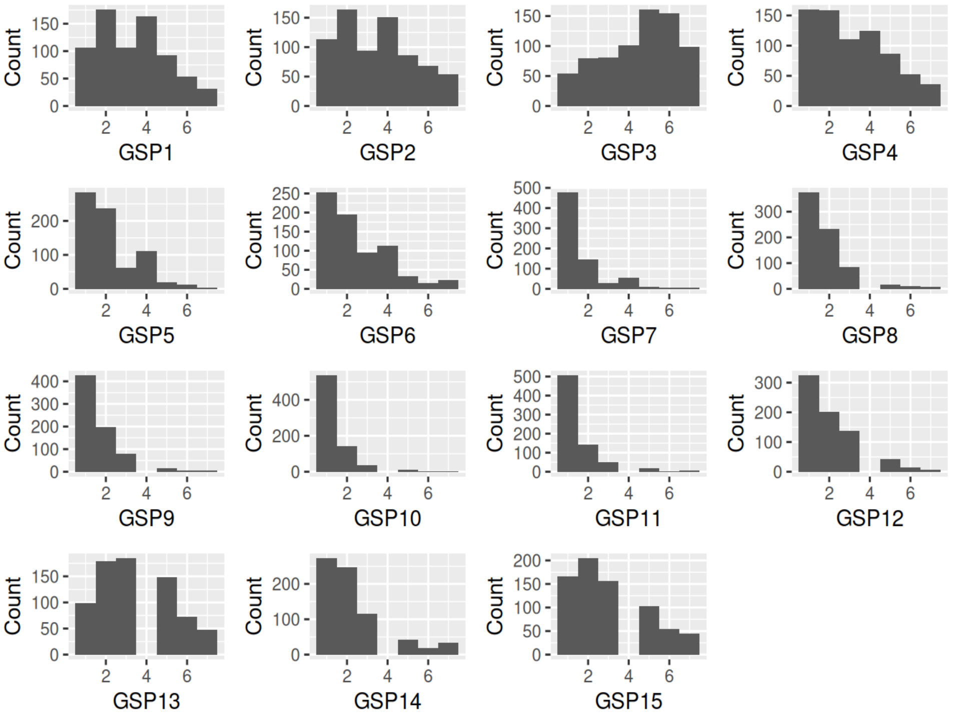

Figure S1. The distribution of responses to the GSP item.

Table S2. Descriptive statistics (*M*, *SD*, Skewness and Curtosis,) as well as Shapiro-Wilk normality test for GSBP item.

Row	*M*	*SD*	*Skewness*	*Kurtosis*	*SW.test*	*p*.value
GSBP1	4.88	1.68	–0.83	–0.01	0.88	<0.001
GSBP2	4.45	1.73	–0.54	–0.56	0.91	<0.001
GSBP3	5.09	1.39	–0.82	0.48	0.9	<0.001
GSBP4	4.44	1.54	–0.37	–0.38	0.94	<0.001
GSBP5	4.80	1.51	–0.54	–0.01	0.92	<0.001
GSBP6	4.56	1.41	–0.41	–0.16	0.94	<0.001
GSBP7	4.66	1.51	–0.46	–0.26	0.93	<0.001
GSBP8	5.21	1.43	–0.57	–0.00	0.91	<0.001
GSBP9	4.47	1.58	–0.36	–0.47	0.94	<0.001
GSBP10	3.53	1.39	0.09	–0.45	0.94	<0.001
GSBP11	2.95	1.41	0.46	–0.42	0.92	<0.001
GSBP12	2.95	1.57	0.57	–0.40	0.91	<0.001
GSBP13	2.48	1.51	0.96	0.22	0.85	<0.001
GSBP14	2.72	1.39	0.67	–0.17	0.9	<0.001
GSBP15	2.64	1.51	0.77	–0.19	0.88	<0.001
GSBP16	2.41	1.48	10.02	0.39	0.84	<0.001
GSBP17	3.04	1.44	0.49	–0.39	0.92	<0.001
GSBP18	3.46	1.62	0.28	–0.63	0.94	<0.001
GSBP19	2.67	1.52	0.75	–0.13	0.88	<0.001
GSBP20	3.18	1.68	0.45	–0.72	0.92	<0.001
GSBP21	2.67	1.54	0.80	–0.07	0.88	<0.001
GSBP22	2.85	1.66	0.69	–0.35	0.89	<0.001

Note: $N = 730$.

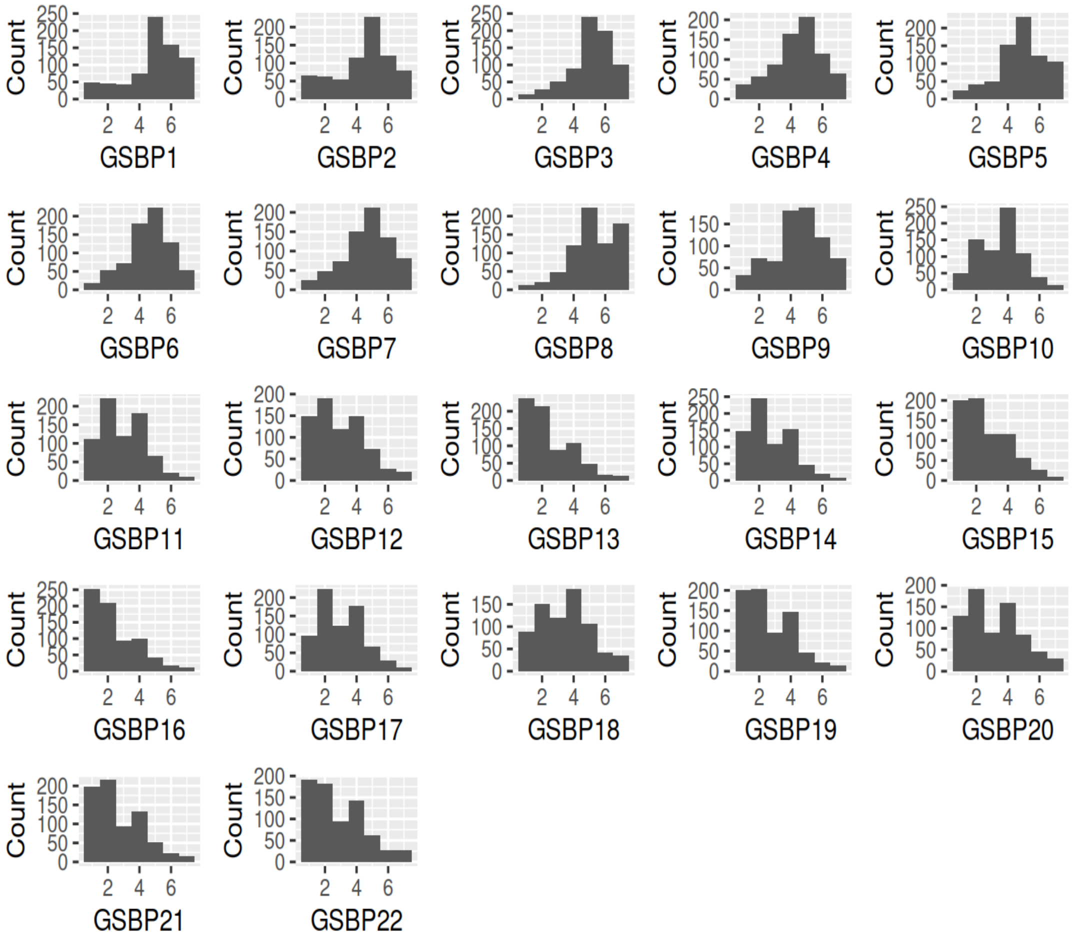

Figure S2. The distribution of responses to the GSBP item.

Table S3. Descriptive statistics (*M*, *SD*, Skewness and Curtosis,) as well as Shapiro-Wilk normality test for IAT item.

Row	*M*	*SD*	*Skewness*	*Kurtosis*	*SW.test*	*p*.value
IAT1	3.11	0.98	–0.25	–0.26	0.90	<0.001
IAT2	2.44	1.09	0.35	–0.66	0.90	<0.001
IAT3	1.41	0.82	2.21	4.67	0.56	<0.001
IAT4	2.02	1.03	0.74	–0.28	0.84	<0.001
IAT5	1.96	0.98	0.86	0.10	0.83	<0.001
IAT6	2.11	1.17	0.80	–0.35	0.83	<0.001
IAT7	2.38	1.14	0.32	–0.94	0.88	<0.001
IAT8	1.90	1.04	1.00	0.24	0.80	<0.001
IAT9	2.04	1.07	0.78	–0.21	0.84	<0.001
IAT10	2.37	1.17	0.36	–0.91	0.88	<0.001
IAT11	1.74	0.97	1.16	0.55	0.75	<0.001
IAT12	1.87	1.05	1.09	0.40	0.78	<0.001
IAT13	1.70	.90	1.13	0.57	0.76	<0.001
IAT14	2.01	1.12	0.89	–0.15	0.81	<0.001
IAT15	1.34	0.71	2.37	5.91	0.54	<0.001
IAT16	2.40	1.18	0.45	–0.74	0.89	<0.001
IAT17	1.86	1.08	1.12	0.43	0.77	<0.001
IAT18	1.47	0.86	1.86	2.78	0.61	<0.001
IAT19	1.51	0.83	1.66	2.27	0.66	<0.001
IAT20	1.41	0.77	2.08	4.16	0.59	<0.001

Note: $N = 730$

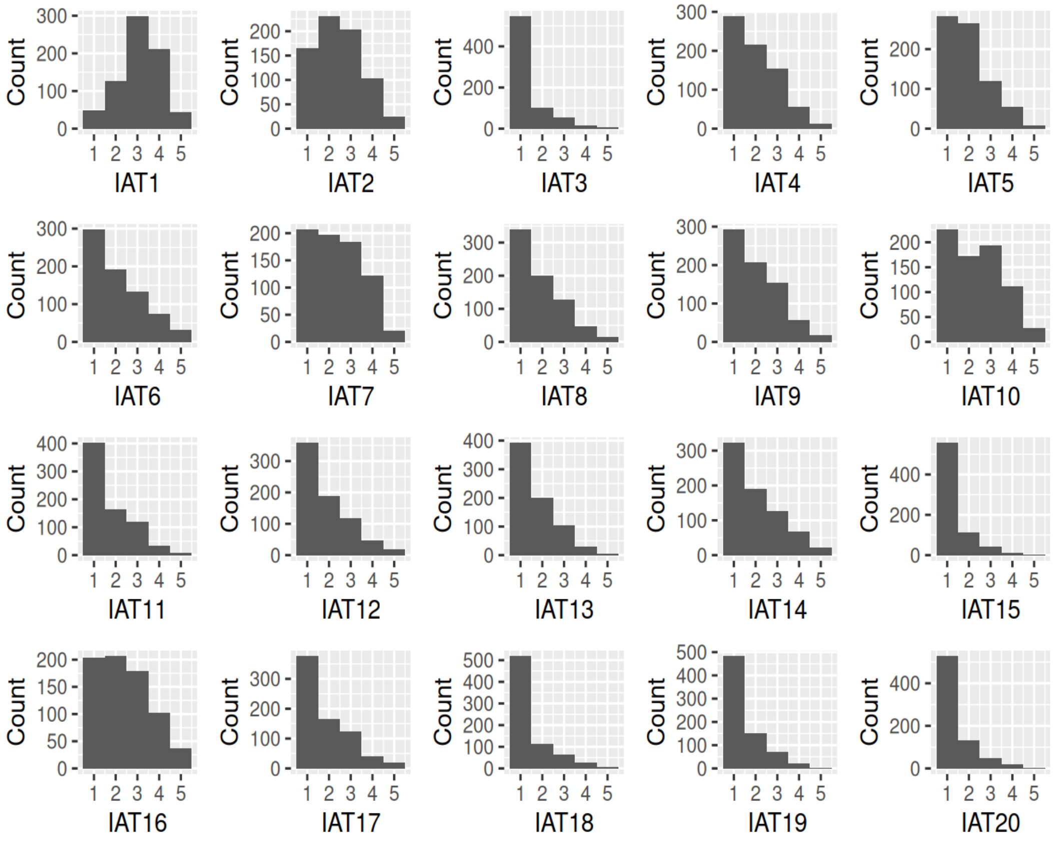

Figure S3. The distribution of responses to the IAT item.

Table S4. Descriptive statistics (*M*, *SD*, Skewness and Curtosis,) as well as Shapiro-Wilk normality test for BC item.

Row	*M*	*SD*	*Skewness*	*Kurtosis*	*SW.test*	*p*.value
BC1	2.46	1.01	–0.01	–1.09	0.88	<0.001
BC2	3.02	1.00	–0.70	–0.61	0.82	<0.001
BC3	1.84	1.01	0.86	–0.55	0.77	<0.001
BC4	1.28	0.67	2.60	6.27	0.47	<0.001
BC5	2.44	0.95	0.02	–0.95	0.88	<0.001
BC6	1.57	0.76	1.22	0.90	0.73	<0.001
BC7	3.22	0.90	–1.01	0.21	0.78	<0.001
BC8	1.47	0.74	1.57	1.85	0.66	<0.001
BC9	2.19	0.88	0.29	–0.67	0.87	<0.001
BC10	2.71	0.93	–0.21	–0.84	0.87	<0.001
BC11	1.21	0.58	3.08	9.48	0.41	<0.001
BC12	2.69	0.93	–0.14	–0.89	0.88	<0.001
BC13	3.26	0.83	–0.97	0.33	0.78	<0.001
BC14	3.31	0.83	–1.11	0.62	0.76	<0.001
BC15	2.67	0.94	–0.14	–0.89	0.88	<0.001
BC16	1.56	0.78	1.33	1.15	0.71	<0.001
BC17	2.94	0.90	–0.40	–0.75	0.85	<0.001
BC18	2.80	0.90	–0.30	–0.71	0.87	<0.001
BC19	2.75	0.97	–0.34	–0.85	0.87	<0.001
BC20	3.24	0.85	–0.96	0.27	0.79	<0.001
BC21	2.86	0.88	–0.38	–0.57	0.86	<0.001
BC22	2.03	1.09	0.60	–1.02	0.81	<0.001
BC23	2.72	0.89	–0.19	–0.74	0.87	<0.001
BC24	2.91	0.88	–0.44	–0.52	0.86	<0.001
BC25	3.21	0.84	–0.82	–0.06	0.8	<0.001
BC26	2.69	0.91	–0.10	–0.87	0.87	<0.001
BC27	2.07	1.09	0.52	–1.09	0.82	<0.001
BC28	1.73	0.86	0.96	0.06	0.78	<0.001

Note: $N = 730$

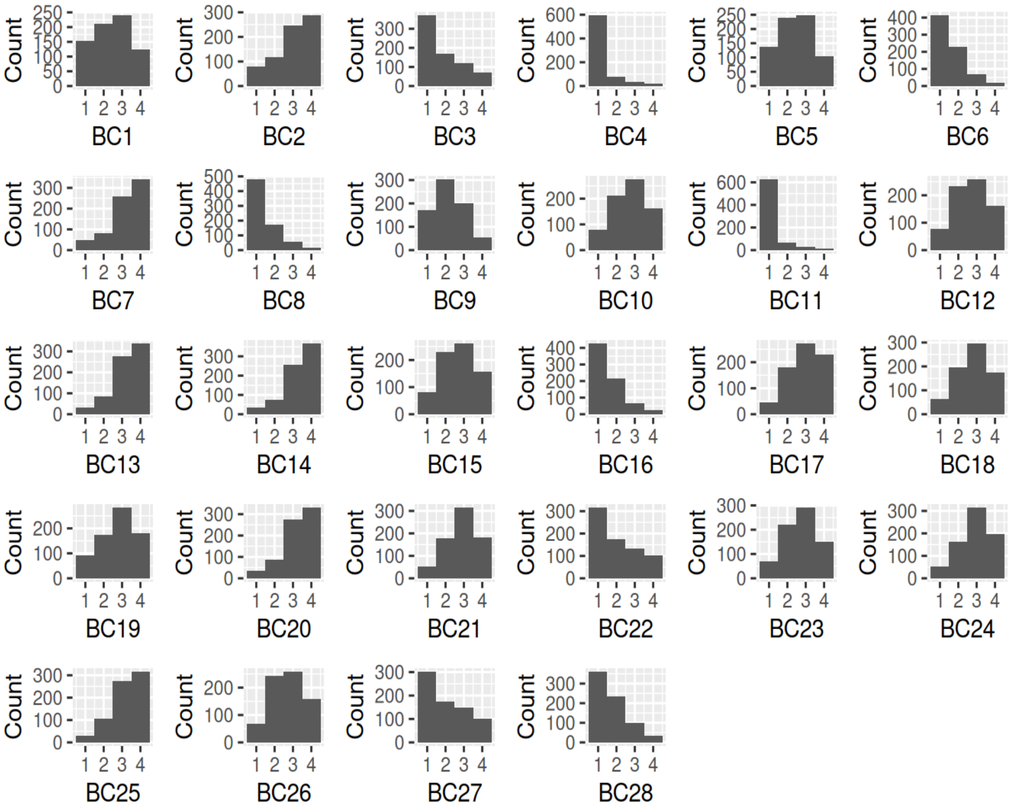

Figure S4. The distribution of responses to the BC item.

Residual Covariances for Manifest variables

Table S5 shows the item residual covariance matrix for the 15 items of the Italian version of the GSP questionnaire.

Table S5. Residual covariance matrix.

	GSP1	*GSP2*	*GSP3*	*GSP4*	*GSP5*	GSP6	GSP7	GSP8	GSP9	GSP10	GSP11	GSP12	GSP13	GSP14	GSP15
GSP1	0.00														
GSP2	0.05	–0.00													
GSP3	0.03	–0.04	0.00												
GSP4	–0.02	–0.06	–0.02	0.00											
GSP5	–0.05	0.04	–0.08	–0.00	0.00										
GSP6	–0.11	0.16	0.03	–0.08	0.08	0.00									
GSP7	–0.00	0.03	0.02	0.04	–0.03	–0.05	–0.00								
GSP8	–0.04	0.08	–0.08	0.02	–0.06	–0.04	0.10	–0.00							
GSP9	–0.02	–0.04	0.03	0.12	–0.02	–0.09	0.12	0.22	–0.00						
GSP10	–0.05	–0.13	–0.06	0.08	–0.06	–0.04	0.10	0.02	0.06	–0.00					
GSP11	–0.07	–0.10	–0.03	0.07	–0.00	–0.08	0.10	0.05	–0.05	0.03	–0.00				
GSP12	–0.03	–0.11	–0.02	0.13	0.08	0.06	0.14	0.13	–0.04	–0.08	0.07	–0.00			
GSP13	0.13	0.09	0.15	0.12	–0.09	–0.02	–0.11	–0.06	–0.06	–0.11	–0.08	0.09	–0.00		
GSP14	–0.12	–0.12	–0.07	–0.02	0.15	0.12	0.12	0.21	0.13	0.04	0.03	0.17	–0.06	0.00	
GSP15	–0.04	–0.08	–0.07	0.10	–0.07	–0.01	–0.03	0.03	0.00	–0.11	–0.06	0.21	0.06	–0.07	–0.00

Table S6 shows the residual covariances among the GSBP items

Table S6. Residual covariance matrix for GSBP.

Row	*GSBP1*	*GSBP2*	*GSBP3*	*GSBP4*	*GSBP5*	GSBP6	GSBP7	GSBP8	GSBP9	GSBP10	GSBP11	GSBP12	GSBP13	GSBP14	GSBP15	GSBP16	GSBP17	GSBP18	GSBP19	GSBP20	GSBP21	GSBP22
GSBP1	–0.00																					
GSBP2	0.12	0.12																				
GSBP3	0.51	0.24	0.00																			
GSBP4	0.34	0.49	0.47	0.00																		
GSBP5	0.02	–0.08	–0.17	–0.05	0.00																	
GSBP6	–0.08	–0.08	–0.11	–0.11	0.15	0.00																
GSBP7	–0.11	–0.09	0.01	–0.10	–0.02	0.03	0.00															
GSBP8	–0.02	–0.12	–0.13	–0.10	0.07	0.03	–0.03	0.00														
GSBP9	–0.11	0.04	0.13	0.16	–0.22	–0.12	0.13	0.07	0.00													
GSBP10	0.05	0.06	0.09	0.22	0.27	0.33	0.24	0.26	0.24	–0.00												
GSBP11	–0.19	–0.01	–0.02	0.18	0.09	0.13	0.08	0.09	0.15	0.18	–0.00											
GSBP12	–0.30	–0.11	–0.08	0.09	0.01	0.02	–0.07	0.03	0.01	0.00	0.00	–0.00										
GSBP13	–0.32	–0.08	–0.18	–0.01	–0.04	–0.01	–0.06	–0.11	0.03	–0.10	–0.04	0.10	–0.00									
GSBP14	–0.15	–0.03	–0.16	0.04	–0.06	–0.03	–0.14	–0.15	–0.10	0.02	0.00	–0.01	0.04	–0.00								
GSBP15	–0.12	–0.02	–0.15	0.01	–0.11	–0.12	–0.18	–0.17	0.00	–0.18	–0.07	0.05	0.01	–0.04	–0.00							
GSBP16	–0.12	–0.07	–0.10	0.04	–0.11	–0.10	–0.12	–0.18	0.01	–0.14	–0.09	–0.05	0.06	–0.04	0.29	–0.00						
GSBP17	0.04	–0.03	–0.03	0.10	0.11	0.15	0.05	0.06	0.03	0.03	0.00	–0.11	–0.08	0.05	–0.01	0.07	–0.00					
GSBP18	–0.11	–0.11	–0.06	0.04	0.16	0.12	0.04	0.12	0.01	0.21	0.02	–0.02	–0.14	–0.02	–0.15	–0.18	0.02	–0.00				
GSBP19	–0.14	–0.19	–0.15	–0.05	0.00	–0.00	–0.14	–0.10	–0.10	0.04	–0.05	–0.16	–0.15	–0.08	–0.13	–0.12	0.02	0.26	–0.00			
GSBP20	–0.01	0.06	–0.05	–0.01	0.02	0.04	–0.05	0.09	0.03	0.10	–0.04	–0.16	–0.16	–0.08	–0.16	–0.11	0.01	0.00	0.06	–0.00		
GSBP21	–0.15	–0.09	–0.15	–0.05	–0.01	0.03	–0.04	0.01	0.05	0.22	0.12	–0.04	0.06	0.14	–0.06	–0.03	0.10	–0.09	–0.06	–0.01	–0.00	5
GSBP22	–0.08	–0.07	–0.14	–0.00	0.06	0.04	–0.02	0.07	0.15	0.24	0.18	0.15	0.18	0.15	0.19	0.11	0.24	–0.22	–0.13	–0.00	0.15	–0.00

AIMS Press

© 2025 the Author(s), licensee AIMS Press. This is an open access article distributed under the terms of the Creative Commons Attribution License (https://creativecommons.org/licenses/by/4.0)
